# Association of Plasma Biomarkers with Sleep Outcomes and Treatment Response After Mild Traumatic Brain Injury

**DOI:** 10.1089/neur.2023.0002

**Published:** 2023-04-12

**Authors:** Shawn R. Eagle, Ava M. Puccio, Denes V. Agoston, Michael Mancinelli, Rachel Nwafo, Peyton McIntyre, Allison Agnone, Savannah Tollefson, Michael Collins, Anthony P. Kontos, Walter Schneider, David O. Okonkwo, Ryan J. Soose

**Affiliations:** ^1^ Department of Neurological Surgery, University of Pittsburgh, Pittsburgh, Pennsylvania, USA.; ^2^ School of Medicine, Uniformed Services University of the Health Sciences, Bethesda, Maryland, USA.; ^3^ Department of Otolaryngology, University of Pittsburgh, Pittsburgh, Pennsylvania, USA.; ^4^ Department of Orthopaedic Surgery, University of Pittsburgh, Pittsburgh, Pennsylvania, USA.; ^5^ Department of Psychology, University of Pittsburgh, Pittsburgh, Pennsylvania, USA.

**Keywords:** blood biomarkers, mild traumatic brain injury, sleep, von Willebrand factor

## Abstract

Sleep disturbances occur in up to 70% of patients with mild traumatic brain injury (mTBI). Modern mTBI management recommends targeted treatment for the patient's unique clinical manifestations (i.e., obstructive sleep apnea, insomnia). The purpose of this study was to evaluate the association of plasma biomarkers with symptom reports, overnight sleep evaluations, and response to treatment for sleep disturbances secondary to mTBI. This study is a secondary analysis of a prospective multiple interventional trial of patients with chronic issues related to mTBI. Pre- and post-intervention assessments were conducted, including overnight sleep apnea evaluation, the Pittsburgh Sleep Quality Index (PSQI), and blinded analysis of blood biomarkers. Bivariate Spearman correlations were conducted for pre-intervention plasma biomarker concentrations and 1) PSQI change scores and 2) pre-intervention sleep apnea outcomes (i.e., oxygen saturation measures). A backward logistic regression model was built to evaluate the association of pre-intervention plasma biomarkers with improvement in PSQI over the treatment period (*p* < 0.05). Participants were 36.3 ± 8.6 years old and 6.1 ± 3.8 years from their index mTBI. Participants reported subjective improvements (PSQI = −3.7 ± 3.8), whereas 39.3% (*n* = 11) had improved PSQI scores beyond the minimum clinically important difference (MCID). PSQI change scores correlated with von Willebrand factor (vWF; ρ = −0.50; *p* = 0.02) and tau (ρ = −0.53; *p* = 0.01). Hyperphosphorylated tau correlated with average saturation (ρ = −0.29; *p* = 0.03), lowest desaturation (ρ = −0.27; *p* = 0.048), and baseline saturation (ρ = −0.31; *p* = 0.02). The multi-variate model (*R*^2^ = 0.33; *p* = 0.001) retained only pre-intervention vWF as a predictor (odds ratio = 3.41; 95% confidence interval, 1.44–8.08; *p* = 0.005) of improving PSQI scores beyond the MCID. vWF had good discrimination (area under the curve = 0.83; *p* = 0.01), with an overall accuracy of 77%, sensitivity of 46.2%, and specificity of 90.0%. Validation of vWF as a potential predictive biomarker of sleep improvement post-mTBI could optimize personalized management and healthcare utilization.

## Introduction

Approximately 53% of patients with mild traumatic brain injury (mTBI) who present to a level 1 emergency department report impairments 1 year post-injury.^1^ Sleep disturbances are among the most common complaints reported after TBI, occurring in up to 70% of patients.^2^ These disturbances can manifest into sleep disorders, such as obstructive sleep apnea (OSA) or insomnia. Modern mTBI management recommends targeted treatment for the patient's unique clinical manifestations. Objective biomarkers to corroborate symptom reports or predict therapeutic response to intervention have not been reported. We sought to evaluate the association of plasma biomarkers with symptom reports, overnight sleep evaluations, and response to treatment for sleep disturbances secondary to mTBI. Past research suggests that higher concentrations of von Willebrand factor (vWF), tau, and hyperphosphorylated tau (p-tau) can be detected in patients with OSA and/or insomnia,^3^ but no study has been conducted in patients with chronic issues from mTBI.

## Methods

### Design and participants

The Targeted Evaluation Action and Monitoring of TBI (TEAM-TBI) study was a prospective multiple interventional trial of patients with secondary sequelae >3 months after mTBI (NCT02657135). Interventions were targeted to the participant's impairments over a 6-month period. Sixty percent of participants (57 of 95) were adjudicated to have a primary or secondary sleep clinical trajectory and were included.^4^ Sleep evaluation consisted of a comprehensive sleep medicine history and physical examination by a physician who was board certified in sleep medicine. Possible interventions included sleep-disordered breathing treatment, cognitive behavioral therapy for insomnia, instruction on sleep hygiene, behavioral regulation strategies, and/or exercise prescription for weight loss. Pre- and post-intervention assessments were conducted, including overnight sleep apnea evaluation, the Pittsburgh Sleep Quality Index (PSQI), and blinded analysis of blood biomarkers (e.g., vWF, tau, p-tau, glial fibrillary acidic protein, brain lipid binding protein, ubiquitin C-terminal hydrolase L1, vascular endothelial growth factor-a, and claudin-5). Biomarkers were chosen before study initiation, based on their empirical association to mTBI and/or common mTBI sequelae. This study was approved by [BLINDED] for human subjects' research.

### Blood biomarker analysis

Plasma samples were analyzed using the reverse-phase protein microarray system. Denatured samples were serially diluted in a 1:2 manner (five-step) and printed onto nitrocellulose film slides by using a Quanterix 2470 Arrayer (Quanterix, Billerica, MA). Slides were dried and blocked with Azure Protein-Free Blocking Buffer (Azure Biosystems, Dublin, CA) and incubated with primary antibodies overnight (8–12 h) at 4°C. After washing, slides were incubated with biotinylated secondary antibodies (1:100,000 dilution). Slides were scanned in an Innopsys InnoScan 710-IR scanner (Innopsys, Carbonne, France) for extended dynamic range signal acquisition at 785 nm. Fluorescence data were imported into a bioinformatics program, and net intensity versus dilution was plotted on a log2-log2 scale. Total amount of antigen was determined by the Y-axis intercept or Y-cept (extrapolating regression line to zero). We express the Y-cept values as log2-transformed Y-cept values, which correspond to the total net intensity of the undiluted plasma sample.

### Sleep apnea testing

Overnight sleep apnea testing was completed with a ResMed ApneaLink four-channel portable monitor (ResMed Corporation, Ponway, CA). Recorded data included airflow, respiratory effort, oximetry, and heart rate, as well as the apnea-hypopnea index and 4% oxygen desaturation index, as defined by the American Academy of Sleep Medicine scoring criteria.^5^

### Validated sleep questionnaire

The PSQI is a validated instrument designed to measure self-reported sleep quality, with global PSQI scores ranging from 0 (no difficulty) to 21 (severe difficulty). A PSQI score of ≤5 is considered normal, and a change of ≥3 is clinically meaningful.^6^

### Statistical analysis

The PSQI was converted into change scores for analysis, where negative scores indicated improvement. Bivariate Spearman correlations were conducted for pre-intervention plasma biomarker concentrations and 1) PSQI change scores and 2) pre-intervention sleep apnea outcome measures. Only statistically significant correlations are reported. A backward logistic regression model was built to evaluate the association of pre-intervention plasma biomarkers with improvement in PSQI over the treatment period (*p* < 0.05). Improvement was defined as change in PSQI score above the minimum clinically important difference (MCID). Discrimination of treatment responders from non-responders using the final model was conducted with the receiver operating characteristic area under the curve (AUC).

## Results

Participants were 36.3 ± 8.6 years old and 6.1 ± 3.8 years from their index mTBI. Approximately 91.2% of the sample was male (*n* = 52), and 75.4% were former military (*n* = 43). Cognitive behavioral therapy for insomnia was the primary indicated treatment (*n* = 18), followed by OSA treatment (*n* = 14), and combination insomnia and OSA management (*n* = 7). The remaining participants did not meet clinical criteria for either disorder and were not assigned a specific treatment pathway in favor of improving sleep hygiene and behavioral regulation (*n* = 18). Approximately 50% of participants returned for a follow-up assessment with complete data (*n* = 28). Of those who returned for follow-up with complete data, 6 were assigned OSA management, 6 were assigned insomnia management, 1 was assigned a combination of OSA/insomnia management, and 15 were not assigned a specific pathway. Participants reported subjective improvements on average (PSQI = −3.7 ± 3.8), whereas 39.3% (*n* = 11) improved PSQI scores beyond the MCID. Participants in the final analysis who were assigned insomnia management improved PSQI scores by the largest magnitude on average (−5.9 ± 4.6), followed by non-specific participants (−3.5 ± 3.4) and OSA participants (−1.7 ± 3.0).

### Bivariate correlation analyses

PSQI change scores correlated with vWF (ρ = −0.50; *p* = 0.02) and tau (ρ = −0.53; *p* = 0.01). Regarding objective testing, tau correlated with apnea index (ρ = −0.36; *p* = 0.01), number of recorded apneas (ρ = −0.28; *p* = 0.03), and obstructive apneas (ρ = −0.33; *p* = 0.01). p-Tau correlated with average saturation (ρ = −0.29; *p* = 0.03), lowest desaturation (ρ = −0.27; *p* = 0.048), and baseline saturation (ρ = −0.31; *p* = 0.02). vWF correlated with average saturation (ρ = −0.32; *p* = 0.02), lowest desaturation (ρ = −0.31; *p* = 0.02), number of minutes with saturation ≤90% (ρ = 0.36; *p* = 0.006), and number of minutes with saturation ≤88% (ρ = 0.40; *p* = 0.002).

### Multi-variate logistic regression model

The model (*R*^2^ = 0.33; *p* = 0.001) retained only pre-intervention vWF as a predictor (odds ratio = 3.41; 95% confidence interval, 1.44–8.08; *p* = 0.005) of improving PSQI scores beyond the MCID. vWF had good discrimination (AUC = 0.83; *p* = 0.01), with an overall accuracy of 77%, sensitivity of 46.2%, and specificity of 90.0%.

## Discussion

In this secondary analysis of a prospective trial for patients with chronic sleep issues related to mTBI, plasma biomarker levels were associated with validated questionnaires, overnight sleep evaluations, and predictive of therapeutic response to targeted intervention. vWF was negatively correlated with PSQI change scores and measures of oxygen saturation and demonstrated good discriminative ability between therapy responders and non-responders. Tau negatively correlated with apnea outcomes, and p-tau was negatively correlated with measures of oxygen saturation.

vWF is a secreted endothelial protein involved in maintaining vascular homeostasis in health and restoring vascular integrity and functionality after noxious stimuli. Elevated vWF plasma levels are indictive of unfavorable outcome after TBI.^7–9^ Higher vWF levels have been reported after sleep disruptions,^10^ short sleep duration, and healthy patients with subjective sleep symptoms.^11^ Though vWF is also associated with sleep apnea, that relationship is mediated by comorbidities (i.e., hypertension) and not the apnea itself.^3^ Multiple studies have reported no change in vWF after use of continuous positive airway pressure (CPAP) therapy.^3^ This may explain why vWF more accurately identified non-responders compared to responders (specificity = 90%).

Tau and p-tau, proteins traditionally associated with neurodegenerative diseases, are also elevated in patients with OSA compared to those without.^12^ In contrast to vWF, tau concentrations decrease after 1 year of CPAP treatment, and even stabilize tau and other clinical assessments in patients with OSA and subjective cognitive impairment.^13^

## Conclusion

Plasma vWF, tau, and p-tau were associated with subjective sleep symptoms and sleep-disordered breathing outcomes (e.g., apnea index, oxygen saturation). vWF was predictive of therapeutic response to sleep therapy after chronic mTBI, accounting for approximately one third of the variance in the outcome and high accuracy in identifying treatment non-responders. Validation of vWF as a potential predictive biomarker of sleep improvement post-mTBI could optimize personalized management and healthcare utilization.

## Abbreviations Used

AUC: area under the curve
CPAP: continuous positive airway pressure
MCID: minimum clinically important difference
mTBI: mild traumatic brain injury
PSQI: Pittsburgh Sleep Quality Index
p-tau: hyperphosphorylated tau
OSA: obstructive sleep apnea
vWF: von Willebrand factor
